# Omission of dexamethasone in prophylaxis for highly emetogenic chemotherapy in patients with breast cancer

**DOI:** 10.31744/einstein_journal/2025AO1124

**Published:** 2025-04-25

**Authors:** Camilla Vieira de Rebouças, Rafaela de Brito Alves, Alayne Magalhães Trindade Domingues Yamada, Auro del Giglio, Felipe José Silva Melo Cruz

**Affiliations:** 1 Núcleo de Pesquisa e Ensino IBCC Oncologia São Paulo SP Brazil Núcleo de Pesquisa e Ensino IBCC Oncologia, São Paulo, SP, Brazil.; 2 Centro Universitário FMABC São Paulo SP Brazil Centro Universitário FMABC, São Paulo, SP, Brazil.

**Keywords:** Dexamethasone, Drug therapy, Nausea, Olanzapine, Vomiting, Breast neoplasms, Antineoplastic agents, Drug-related side effects and adverse reactions

## Abstract

**Objective:**

Chemotherapy-induced nausea and vomiting are highly prevalent adverse events that can lead to poor treatment adherence and a decreased quality of life. To the best of our knowledge, the complete omission of dexamethasone from any regimen for preventing nausea and vomiting has not yet been evaluated. This study aimed to evaluate the efficacy of a three-drug protocol without corticosteroids for preventing nausea and vomiting.

**Methods:**

This prospective, single-arm, phase II study was designed to evaluate the efficacy of olanzapine, netupitant, and palonosetron in controlling nausea and vomiting induced by emetogenic chemotherapy. Patients were assigned to receive olanzapine on days 1-5 and netupitant and palonosetron on day 1. No corticosteroids were administered. The primary endpoint was complete nausea control during the first 5 days after chemotherapy. Secondary endpoints included complete emesis control (no emesis and no use of rescue medication) and overall complete control (no emesis, no rescue medication, and no nausea).

**Results:**

The complete nausea control rate was 46% (95% confidence interval [95%CI] 0.32-0.59). The emesis control rate was 68% (95%CI= 0.55-0.80), and the overall control rate was 46% (95%CI= 0.32-0.59).

**Conclusion:**

These findings suggest that omitting dexamethasone in highly emetogenic chemotherapy is feasible and results in nausea and vomiting control rates similar to those of the standard four-drug protocol. However, randomized controlled trials are required to confirm this hypothesis.

## In Brief

A phase II study evaluated a corticosteroid-free regimen (olanzapine, netupitant, and palonosetron) for the treatment of chemotherapy-induced nausea and vomiting. The results showed control rates comparable to those of standard protocols, demonstrating its feasibility without dexamethasone.

## Highlights

■ Evaluation of a corticosteroid-free antiemetic regimen.

■ Primary endpoint: 46% nausea control.

■ Secondary endpoint: 68% emesis control.

■ Comparable to standard four-drug protocols.

## Graphical abstract

**Figure:**
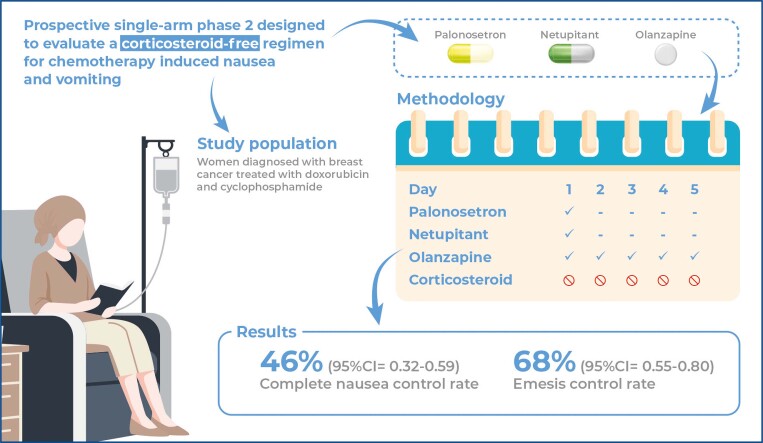

## INTRODUCTION

Chemotherapy-induced nausea and vomiting are highly prevalent adverse events^1-3^ that can lead to a decreased quality of life, dose reductions, and treatment interruptions.^4^ Treatment protocols are classified as highly, moderately, or low emetogenic chemotherapy.^5^ Combination of anthracycline and cyclophosphamide (AC) has been the backbone of treatment of different breast cancer scenarios and, in many cases, remains the standard of care.^6^ Anthracycline and cyclophosphamide is associated with an up to 83% risk of nausea and vomiting and is therefore considered highly emetogenic.^7-9^

The current standard of care for highly emetogenic chemotherapy (HEC) is a four-drug regimen consisting of olanzapine,^10^ a 5-hydroxytryptamine type 3 receptor (5HT3) antagonist, a neurokinin 1 receptor (NK1) antagonist, and dexamethasone.^2,11-13^ This highly effective protocol can achieve up to 60% complete control of emesis.^10^

The first NK1 antagonist (aprepitant) was approved in 2004.^9^ Until then, the recommended scheme for nausea prevention consisted of dexamethasone plus a 5HT3 antagonist (ondansetron or palonosetron),^14^ with or without the dopamine antagonist metoclopramide.^11^ In 2014, a fixed-dose oral combination of netupitant and palonosetron, combined with dexamethasone, was approved for preventing chemotherapy-associated nausea and vomiting.^15^ Olanzapine was added to the 2017 guidelines for HEC^16^ after a positive phase III trial was published in 2016.^10^

Corticosteroids have been used for decades to prevent nausea associated with chemotherapy and radiotherapy.^8^ Their antiemetic effects are attributed to the reduced production of inflammatory mediators, such as eicosanoids, inhibiting serotonin production, and modulating the hypothalamic-pituitary-adrenal axis.^17^ However, corticosteroids also affect lipid and glucose metabolism, bone maintenance, immune system regulation, memory, mood, and sleep balance. Therefore, they are associated with side effects such as insomnia, weight gain, and mood disorders.^18-20^

Avoiding dexamethasone may also benefit patients receiving concurrent immunotherapy, as steroids have the potential to reduce immunotherapy effectiveness.^21^ To our knowledge, the complete omission of dexamethasone from nausea prevention protocols for HEC has not been evaluated previously.^8, 22^

## OBJECTIVE

This study aimed to evaluate the efficacy of a three-drug protocol without corticosteroids for preventing nausea and vomiting.

## METHODS

This prospective, single-arm, phase II study was designed to evaluate the efficacy of olanzapine, netupitant, and palonosetron in controlling HEC-induced nausea and vomiting. The trial was conducted in accordance with the principles of the Declaration of Helsinki and was approved by the Ethics Committee of *Instituto Brasileiro de Controle do Cancer* (CAAE: 38285020.8.0000.0072; #4.483.028). Eligible patients were women with histologically confirmed breast cancer who were scheduled to start treatment with doxorubicin and cyclophosphamide. Exclusion criteria included the use of opioids or antipsychotic medications, the presence of medical conditions that could potentially cause vomiting, and the inability to take oral medications. Informed consent was obtained from all participants. Patients were assigned to receive olanzapine (5mg once daily) on days 1-5 and netupitant (300mg) with palonosetron (0.5mg) on day 1. No corticosteroids were administered. Outcomes were analyzed using the classic visual analog scale,^23^ which was completed by the patients during the first 5 days of treatment. Additionally, a questionnaire was used to collect sociodemographic information.

The primary endpoint was complete control of nausea during the first 5 days after chemotherapy administration. Secondary endpoints included complete emesis control (no emesis and no use of rescue medication for the first 120h), complete control (no emesis, no nausea, and no rescue medication for the first 120h), and rates of acute and delayed nausea. Acute nausea was defined as nausea occurring within the first 24h after treatment, whereas delayed nausea referred to nausea occurring within the first 5 days after chemotherapy.

### Statistical analysis

Summary statistics are presented as frequencies and proportions for categorical data and as means and ranges for continuous variables.

The null hypothesis considered that a regimen containing olanzapine, netupitant, and palonosetron would not effectively control nausea. Based on a previous phase III study of olanzapine,^24^ we set a nausea control rate of 20% and expected control rate of 40% for the present study. To achieve a 5% significance level (two-sided) and 80% statistical power, we calculated a minimum sample size of 50 patients, assuming a 5% dropout rate. A one-sample test of proportion was used to analyze the data based on a per-protocol analysis.

## RESULTS

**Figure 1:**
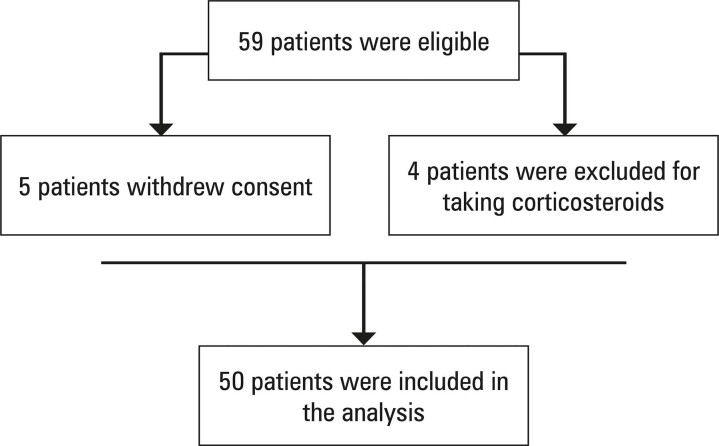
Study selection of participants

Of 59 patients eligible for participation in the study, we excluded 5 patients who withdrew consent and 4 who took corticosteroids against the recommendation. Therefore, 50 patients were enrolled between January 2020 and December 2021 (Figure 1).

The mean age of the study population was 47.6 years (range, 29-78 years). A minority of patients (4%) received chemotherapy with palliative intent, whereas 96% received chemotherapy with curative intent. Ten patients (20%) reported a history of smoking, and only one patient (2%) reported a history of alcohol abuse. A summary of the baseline characteristics is presented in table 1.

**Table 1:** Demographic and clinical characteristics of the study patients

Age, year	
Mean	47.6
Range	29-78
Race, n (%)	
White	26 (52)
Black	21 (42)
Asian	2 (4)
Hispanic	1 (2)
Education level, n (%)	
No formal education	2 (4)
Elementary school	7 (14)
Intermediate school	12 (24)
High school	16 (32)
Graduate school	11 (22)
Postgraduate	2 (4)
Chemotherapy intent, n (%)	
Curative	48 (96)
Palliative	2 (4)
Smoking History, n (%)	
No	40 (80)
Yes	10 (20)
Alcohol Abuse History, n (%)	
No	49 (98)
Yes	1 (2)

For the primary endpoint, the complete nausea control rate was 46% (95%CI= 0.32-0.59). The acute and delayed nausea control rates were both 52% (95%CI= 0.38-0.65; Table 2). For the secondary endpoints, the emesis control rate was 68% (95%CI= 0.55-0.80), and the complete control rate was 46% (95%CI= 0.32-0.59) (Table 3). One patient dropped out due to grade 2 dizziness and drowsiness following the administration of olanzapine but was included in the final analysis.

**Table 2:** Nausea control rate

	Total	95%CI
0-24h after chemotherapy (acute), n (%)		
No nausea	26 (52)	0.38-0.65
Nausea	24 (48)	
24-120h after chemotherapy (delayed), n (%)		
No nausea	26 (52)	0.38-0.65
Nausea	24 (48)	
0-120h after chemotherapy (overall), n (%)		
No nausea	23 (46)	0.32-0.59
Nausea	27 (54)	

**Table 3:** Emesis, overall nausea, and vomiting control

	Total	95%CI
Emesis control 0-120 h after chemotherapy, n (%)		
No emesis	34 (68)	0.55-0.80
Emesis	16 (32)	
Overall nausea and vomiting control rate, n (%)		
No nausea/no emesis	23 (46)	0.32-0.59
Nausea/emesis	27 (54)	

## DISCUSSION

This trial provides a new perspective on nausea and vomiting prevention as to our knowledge, no trial has attempted to exclude corticosteroids from protocols for preventing chemotherapy-induced nausea and vomiting.^2,10,25^ This approach is particularly interesting for patients with specific or relative contraindications to corticosteroid use, such as diabetes, a history of gastric ulcer, osteoporosis, tuberculosis, and glaucoma. Moreover, excluding corticosteroids could minimize weight gain, insomnia, immunosuppression, and edema.^25,26^ A phase II trial assessed the adverse effects of dexamethasone use in chemotherapy-induced nausea and vomiting prophylaxis and found that insomnia was the most frequent adverse event, followed by gastric discomfort.^26^ Owing to the high rate of reported symptoms, a controlled trial was suggested to evaluate the feasibility of a non-steroid protocol for preventing chemotherapy-induced nausea and vomiting.

We propose a protocol for controlling chemotherapy-induced nausea and vomiting that completely omits corticosteroids. One non-inferiority trial evaluated a dexamethasone-sparing regimen, comparing 12mg of dexamethasone administered only on day 1 to 12mg administered on days 1-4. The protocol also included netupitant and palonosetron but did not include olanzapine. The non-inferiority endpoint was satisfied.^27^ The no-steroid protocol proposed in this study-which used olanzapine, netupitant, and palonosetron-achieved an overall nausea control rate of 46%. This finding is similar to that reported by Navari et al.^10^ in a study that described and validated a four-drug regimen, which has become the gold standard in the prevention of chemotherapy-induced nausea and vomiting. Similar studies involving olanzapine have also demonstrated its efficacy.^12,13,28^ A published meta-analysis concluded that adding olanzapine could reduce the incidence of nausea from 75% to 50% in chemotherapy-induced nausea and vomiting.^29^

We chose to use olanzapine at a 5 mg daily dose, half the dose used in the trial described by Navari et al.^10^ This dose reduction has already been proposed and validated by guidelines and previous studies to prevent sedation caused by the drug.^12,30^ Nevertheless, one dropout due to drowsiness and sleepiness occurred in our study. A trial published in 2024 evaluated lower doses of olanzapine (2.5mg) for emesis prophylaxis and achieved a non-inferior endpoint.^31^

One limitation of this study is that we included only patients with breast cancer receiving doxorubicin and cyclophosphamide, limiting the generalizability of our findings to patients with other cancers or those receiving different chemotherapy regimens. A randomized controlled phase III trial, including a broader population, is necessary to validate the complete omission of corticosteroids in preventing chemotherapy-induced nausea and vomiting.

## CONCLUSION

Omitting dexamethasone for highly emetogenic chemotherapy is feasible and shows similar control of nausea and vomiting to that of the standard four-drug protocol. Further phase III controlled studies are needed to validate this protocol as a potential prophylactic regimen of choice for patients with contraindications for dexamethasone use.

## STATEMENTS AND DECLARATIONS

Felipe José Silva Melo Cruz has served on advisory boards for Novartis and Janssen and has received travel support from Janssen.
